# Insights into the Role of the Discontinuous TM7 Helix of Human Ferroportin through the Prism of the Asp325 Residue

**DOI:** 10.3390/ijms22126412

**Published:** 2021-06-15

**Authors:** Marlène Le Tertre, Ahmad Elbahnsi, Chandran Ka, Isabelle Callebaut, Gérald Le Gac

**Affiliations:** 1 Univ Brest, Inserm, EFS, UMR 1078, GGB, F-29200 Brest, France; marlene.letertre@gmail.com (M.L.T.); chandran.ka@chu-brest.fr (C.K.); 2 CHRU de Brest, F-29200 Brest, France; 3 Sorbonne Université, Muséum National d’Histoire Naturelle, UMR CNRS 7590, Institut de Minéralogie, de Physique des Matériaux et de Cosmochimie, IMPMC, F-75005 Paris, France; elbahnsi@ymail.com (A.E.); isabelle.callebaut@sorbonne-universite.fr (I.C.); 4 Laboratory of Excellence GR-Ex, F-75015 Paris, France

**Keywords:** ferroportin, major facilitator family, extracellular gate dynamics, gating helix, iron export mechanism

## Abstract

The negatively charged Asp325 residue has proved to be essential for iron export by human (HsFPN1) and primate Philippine tarsier (TsFpn) ferroportin, but its exact role during the iron transport cycle is still to be elucidated. It has been posited as being functionally equivalent to the metal ion-coordinating residue His261 in the C-lobe of the bacterial homolog BbFpn, but the two residues arise in different sequence motifs of the discontinuous TM7 transmembrane helix. Furthermore, BbFpn is not subject to extracellular regulation, contrary to its mammalian orthologues which are downregulated by hepcidin. To get further insight into the molecular mechanisms related to iron export in mammals in which Asp325 is involved, we investigated the behavior of the Asp325Ala, Asp325His, and Asp325Asn mutants in transiently transfected HEK293T cells, and performed a comparative structural analysis. Our biochemical studies clearly distinguished between the Asp325Ala and Asp325His mutants, which result in a dramatic decrease in plasma membrane expression of FPN1, and the Asp325Asn mutant, which alters iron egress without affecting protein localization. Analysis of the 3D structures of HsFPN1 and TsFpn in the outward-facing (OF) state indicated that Asp325 does not interact directly with metal ions but is involved in the modulation of Cys326 metal-binding capacity. Moreover, models of the architecture of mammalian proteins in the inward-facing (IF) state suggested that Asp325 may form an inter-lobe salt-bridge with Arg40 (TM1) when not interacting with Cys326. These findings allow to suggest that Asp325 may be important for fine-tuning iron recognition in the C-lobe, as well as for local structural changes during the IF-to-OF transition at the extracellular gate level. Inability to form a salt-bridge between TM1 and TM7b during iron translocation could lead to protein instability, as shown by the Asp325Ala and Asp325His mutants.

## 1. Introduction

Ferroportin 1 (FPN1; also known as SLC40A1) is the sole iron export protein reported in mammals and is considered to be a key player in both cellular and systemic iron homeostasis. It is expressed in all types of cells that handle major iron flow: macrophages, duodenal enterocytes, hepatocytes, and placental syncytiotrophoblasts [1]. Plasma membrane expression is predominantly regulated by the liver-derived peptide hepcidin, which induces internalization and degradation of FPN1 and thereby decreases iron delivery to plasma [2,3]. The hepcidin–ferroportin axis plays an important role in the pathogenesis of inherited and acquired iron metabolism disorders such as hemochromatosis, β-thalassemia, or iron-restrictive anemia [4,5]. Downregulation of FPN1 has also been detected in various types of cancer, where it is thought to promote iron retention and cell proliferation [6].

FPN1 belongs to the Major Facilitator Superfamily (MFS), a large family of secondary active transporters that control the flow of a wide range of substrates (inorganic ions, metabolites, neurotransmitters, and drugs) across biological membranes [7,8,9,10]. Despite low sequence identities, MFS members share a common architecture comprising two bundles, each including 6 transmembrane (TM) helices (N-lobe: TM1-TM6; C-lobe: TM7-TM12), related by twofold pseudosymmetry. The two bundles interact together and orchestrate transitions between two extreme conformational states: IF and OF. A common “alternating access” model, which is shared with other solute carrier (SLC) transporters, was proposed over 50 years ago to account for substrate translocation across biological membranes [11]. According to this model, a membrane transporter undergoes large conformational changes that alternately expose the substrate to one side of the membrane or the other. During the structural transitions, the substrate sits at the approximate center of the transporter and is not accessible to either side of the membrane.

This “alternating access” model has been refined in recent years to better reflect the specific characteristics of MFS proteins within the lipid bilayer [9,10,12]. The OF conformation can be converted to IF, and vice versa (IF to OF), by concerted conformational changes in the two lobes over a rotation axis that crosses a central-binding site and involves occluded intermediates. The transitions between the different states involve not only rigid-body rotations of the N- and C-lobes, in accordance with the “rocker-switch” model [12,13], but also plasticity at the level of individual transmembrane helices. In the more recent “clamp-and-switch” model [9], it is suggested that occlusion of the binding site from the cytoplasmic side is mainly achieved through bending the cytoplasmic ends of helices TM4 and TM10, and occasionally the flanking helices TM5 and TM11, whereas occlusion from the extracellular side is mainly achieved through bending the extracellular ends of helices TM1 and TM7 and flanking TM2 and TM8 helices. The conformational transitions are orchestrated by the formation and disruption of non-covalent inter- and intra-domain interactions, among which salt-bridges are particularly important [9,10,14].

Since 2015, several 3D structures of ferroportin have been captured in different organisms and in different conformational states, with various ions mimicking the cognate substrate in the binding sites. These structures offer us the opportunity to better understand the molecular basis of the iron transport cycle and to identify mammalian or human specificities, and in particular those related to hepcidin regulation. Taniguchi et al. were the first to use X-ray crystallography to solve the 3D structures, in both the OF and IF conformations, of a bacterial homologue of the iron exporter (Bd2019; now referred to as BbFpn). The authors confirmed the typical MFS fold, which was previously anticipated based on sequence analysis [15,16,17], but also revealed a distinct structural feature in TM7: a non-helical stretch of 6 residues at its center separates the helix into in a longer TM7a and a shorter TM7b. They highlighted the importance of TM7b for the flexibility of the C-lobe and the formation of the extracellular gate in the IF state [18]. Deshpande et al. then reported the crystal structure of Ca^2+^-bound BbFpn in an IF conformation and argued that human FPN1 iron transport activity is dependent on extracellular Ca^2+^, which, they suggested, binds in a N-lobe pocket, while Fe^2+^ was expected to be in the C-lobe (as suggested by the binding of nickel in presence of EDTA in the unwound TM7 region). The authors suggested an iron efflux mechanism whereby TM7b participates in the metal-binding site in the IF conformation (when the iron exporter is open to the cytoplasm), and then moves away to promote iron release while ferroportin returns to its basal OF conformation (when the iron exporter is open to the extracellular milieu) [19]. BillesbØlle et al. recently used cryo-electron microscopy to solve OF structures of human FPN1 (HsFPN1) in lipid nanodiscs, both in apo state and in the presence of hepcidin and cobalt. The authors settled the question of Fe^2+^ binding site(s) by highlighting the presence of Co^2+^ in both the N- and C-lobes, with possible coupling of the latter to hepcidin in a way that could also be dependent of the mobility of TM7b [20]. The same cryo-electron microscopy technique was very recently used by Pan et al. to solve the structures of ferroportin in the primate Philippine Tarsier (TsFpn) in an OF state, in the presence and absence of hepcidin. The authors confirmed the existence of two metal ion (Co^2+^)-binding sites. Using proteoliposomes, they further showed that Fe^2+^ export is coupled to transport of H^+^ [21].

The discontinuous TM7 transmembrane helix thus appears to play a central role in the step-by-step mechanism of iron export, with subtle differences between species. This is well illustrated by His261 in the C-lobe of BbFpn and the Asp325 residue, thought to be functionally equivalent, in HsFPN1 and TsFpn. In BbFpn, His261, which is located in the C-terminal extremity of the unwound TM7 region at the base of TM7b, has been proved to be a critical residue for metal binding [18,19]. Molecular dynamic (MD) simulations of the currently available structure of human FPN1 in the OF state showed that Asp325, located at the same position as BbFpn His261, was localized in the close vicinity of Fe^2+^, together with Thr320 and Cys326 and residues from TM11 (Asp504 and His507), forming a site that may play a regulatory role. Another iron-binding site was identified in the N-lobe, which could be more directly related to the iron export function [20]. This hypothesis is supported by biochemical studies demonstrating that: (i) mutation of Asp39 to alanine in the N-lobe metal-binding site of HsFPN1 completely abolishes iron egress [16]; (ii) mutations of Asp325 (Asp to Ala, His, or Asn) in the C-lobe metal-binding site of HsFPN1 also drastically reduce iron efflux [16,19], but not mutations of Cys326 (Cys to Ser, Phe, or Tyr) [17,20,22] or mutation of His507 to arginine [23] or of Asp504 to Asn [24]; and (iii) the Cys326 and His507 mutants are resistant to downregulation by hepcidin [17,20,22,23,24]. It is also supported by MD simulations of BbFpn in an IF state with an excess of Ni^2+^ ions [19], suggesting that the N-lobe is a primary and conserved iron-recognition site.

In the present study, we differentiated Asp325Ala, His, and Asn mutants according to cell surface expression and not just their ability to export iron. We correlated our observations to a comparative analysis of the 3D structures of HsFPN1 and TsFpn in the OF state, excluding a direct interaction between Asp325 and Co^2+^. Based on the possible arrangement of the HsFPN1 and TsFpn N- and C-lobes in the IF state, we suggest that Asp325 may form an inter-lobe salt-bridge with Arg40 (TM1) when not interacting with Cys326. This interaction may be a key feature of the extracellular gate, being involved in the conformational switch when iron sits in the core of the transporter awaiting translocation.

## 2. Results

Obvious deleterious effects on the ability of human FPN1 to export iron were reported for the Asp325Ala (two groups) [16,19], and p.Asp325His and p.Asp325Asn (one group) amino acid substitutions [19]. The impact of the three mutants on the cell surface expression of HsFPN1 was less clear. This led us to re-evaluate the effects of the three mutants, using different protocols, and to further investigate the structure-specific characteristics of the negatively charged Asp325 residue in both the OF and IF conformations.

### 2.1. Effects of the Asp325Ala, His, and Asn Mutants on the Cell Surface Expression of Human FPN1 and Its Ability to Export Iron

**Figure 1:**
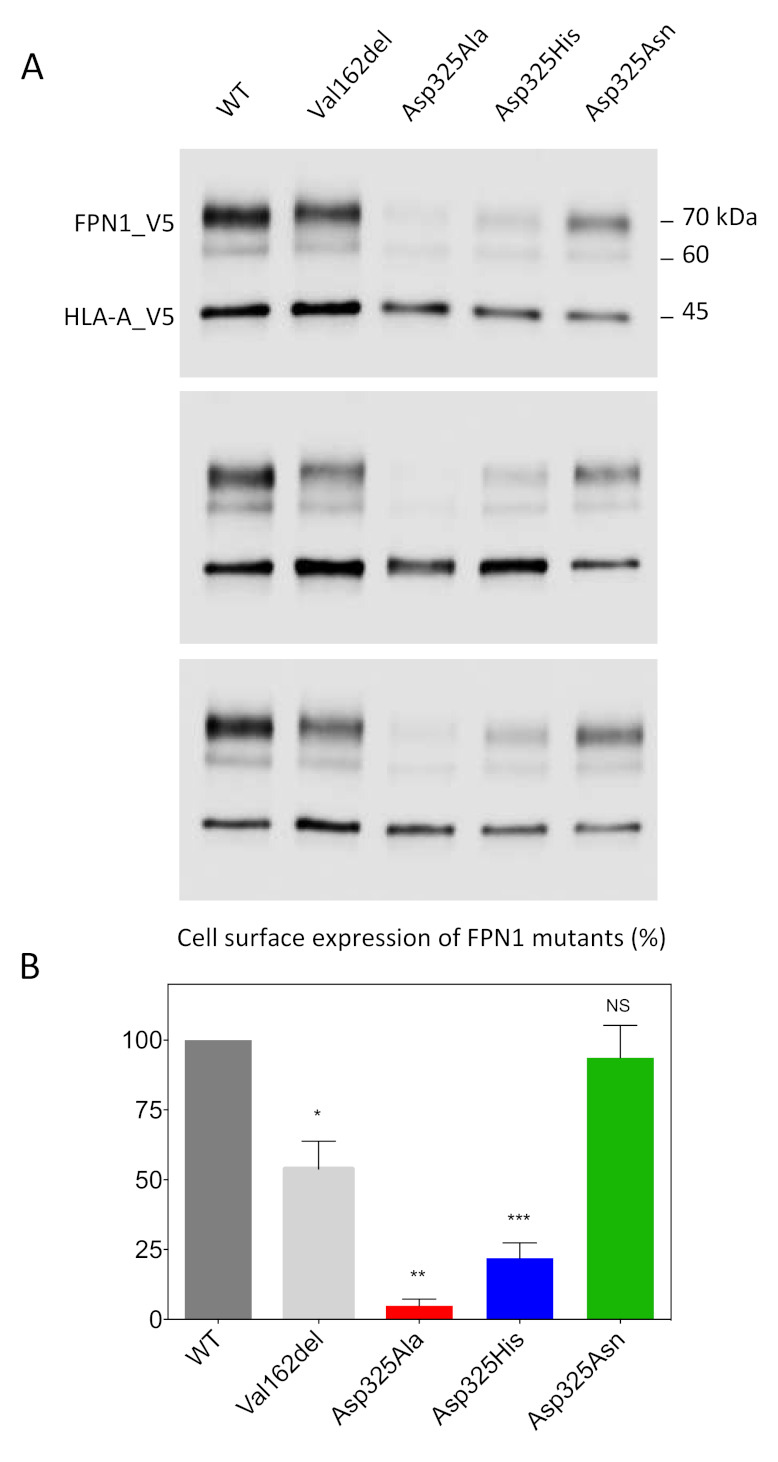
Effects of the Asp325Ala, His, and Asn variants on the cell-surface expression of HsFPN1. (**A**) HEK293T cells were transiently co-transfected with plasmids encoding either a V5-tagged FPN1 protein (WT or variant) or a V5-tagged HLA-A protein. Human leukocyte antigen (HLA)-A was used as a control and as a standard for normalization, being a cell-surface protein with no known role in iron metabolism. At 48 h after transfection, cell-surface proteins were selectively purified and analyzed by Western blotting using a peroxidase-conjugated mouse anti-V5 antibody. (**B**) Densitometric scans of FPN1 levels normalized to HLA-A. The error bars represent the standard deviation of the 3 independent experiments presented in panel A. *p* values were calculated using Student’s *t*-test. * *p* < 0.05, ** *p* < 0.01, and *** *p* < 0.001.

We used a cell-surface biotinylation method to separate proteins on the plasma membrane from those in the intracellular compartments. Cultured HEK293T cells were transiently co-transfected with plasmids encoding FPN1-V5 fusion proteins or the Human Leukocyte Antigen HLA-A (also fused to a V5 epitope tag). The Val162del mutant, which is known to decrease cell-surface localization, was used as a negative control. Twenty-four hours after transfection, the membrane proteins were biotinylated, purified, and analyzed by Western blot and densitometry (Figure 1). HLA-A was used as a control and as a standard for normalization because it is a cell-surface protein with no known role in iron metabolism. As expected, the expression of WT FPN1 resulted in cell-surface localization, while the Val162del mutant showed markedly reduced localization. The Asp325Asn mutant did not cause obvious mislocalization of the iron exporter, in contrast to the Asp325Ala and Asp325His mutants, which were only weakly detected.

**Figure 2:**
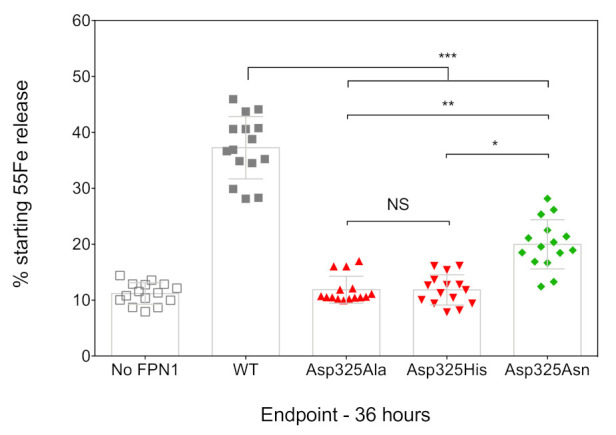
Effects of the Asp325Ala, His, and Asn variants on the iron export function of HsFPN1. HEK293T cells were transiently transfected with WT or mutated FPN1-V5 expression plasmids for 24 h before being grown in 20 µg/mL ^55^Fe-transferrin. After 16 h, cells were washed and then serum-starved for up to 36 h. Data are presented as percentage cellular radioactivity at time zero. The error bars represent the standard deviation of 5 independent experiments (performed in triplicate; n = 15). *p* values were calculated using Student’s *t*-test. * *p* < 0.05, ** *p* < 0.01, and *** *p* < 0.001.

The ability of the FPN1 mutants to export iron out of the cell was investigated using radiolabeled transferrin-bound iron. Cultured HEK293T cells were transiently transfected with plasmids encoding FPN1-V5 fusion proteins, grown in 20 µg/mL ^55^Fe-transferrin for 16 h, washed, and placed in serum-free medium. The amount of ^55^Fe exported into the supernatant was measured after 36 h, using cells transfected with the commercial pcDNA3.1-V5-His vector as a negative control (no FPN1). As shown in Figure 2, the cells transfected with the WT FPN1-V5 construct displayed a threefold increase in iron release. None of the tested Asp325 mutants were able to cause iron release in amounts comparable to the WT FPN1 (*p* < 0.001). The observed differences were similar to those reported by Deshpande et al., with massive reductions in iron efflux for the Asp325Ala and Asp325His mutants, and a considerably smaller effect of the Asp325Asn mutant (325Asn vs. 325His: *p* < 0.05; 325Asn vs. 325Ala: *p* < 0.01).

### 2.2. 3D Structure Analysis

**Figure 3:**
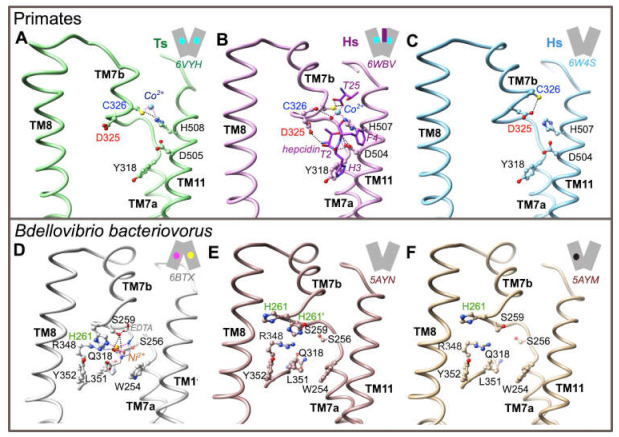
Environment of the ion-binding site at the level of C-lobe TM7b in both the BbFpn and HsFPN1/TsFpn 3D structures. All the considered 3D structures are in an outward-facing conformation (**A**–**C**, **E**, **F**), except for BbFpn in complex with Ni^2+^ and EDTA (inward-facing state-**E**). PDB codes are indicated for each of the represented structures. Two possible conformers are shown for His261 in the BbFpn 3D structure in panel E. H-bonds and salt-bridges are shown with dashed lines.

In order to get further insight into the role of Asp325 during the iron transport cycle, we first analyzed 3D structures in primate proteins solved in the OF state (Figure 3; panels A to C). Asp325 is not a direct ligand of the metal, either in TsFpn in presence of Co^2+^ (pdb 6VYH, Figure 3A; [21]), or in HsFPN1 in presence of Co^2+^ and hepcidin (pdb 6WBV, Figure 3B; [20]). Cys326 and His508(TsFpn)/His507(HsFPN1) are directly involved in the coordination of the metal, with atoms from the N- and C-terminus of hepcidin and water molecules providing additional ligands in the ferroportin-hepcidin complex to fulfill a tetrahedral coordination geometry (Figure 3B). In the HsFPN1-hepcidin complex, Asp325 is linked through its main-chain carbonyl oxygen to a water molecule, which is itself involved in metal coordination. In contrast, in the TsFpn 3D structure (not showing a tetrahedral coordination geometry of the metal), Asp325 is free (Figure 3A). In the HsFPN1 3D structure without metal and without hepcidin (pdb 6W4S, Figure 3C; [20]), Asp325 forms a hydrogen bond with Cys326. Thus, Asp325, unlike His261 in the bacterial exporter, appears not to play a role in direct coordination of a metal (at least, Ni^2+^ as observed in the complex considered in Figure 3D (pdb 6BTX) [19]), but rather provides a specific local environment allowing regulation of the metal-binding site. It is noteworthy that the metal is positioned differently at the base of TM7b in BbFnp and in primate TsFpn/HsFPN1: nickel is positioned within the pocket formed by the discontinuous TM7 in BbFnp (IF state; Figure 3D) whereas, in primate TsFpn/HsFPN1 (Figure 3A,B), cobalt binds deeper in the C-lobe, between TM7b and TM11. This difference would not be due to different binding patterns following the considered divalent metals but reflects specific features of primate versus bacterial transporters. In the OF state, when not involved in metal coordination (Figure 3E (apo, pdb 5AYN) and Figure 3F (Fe^2+^ bound in the N-lobe (pdb 5AYM)) [18], the BbFpn His261 side-chain is rotated at 180°, similarly to what is observed for TsFpn Asp325 when not interacting with Cys326 (Figure 3A).

**Figure 4:**
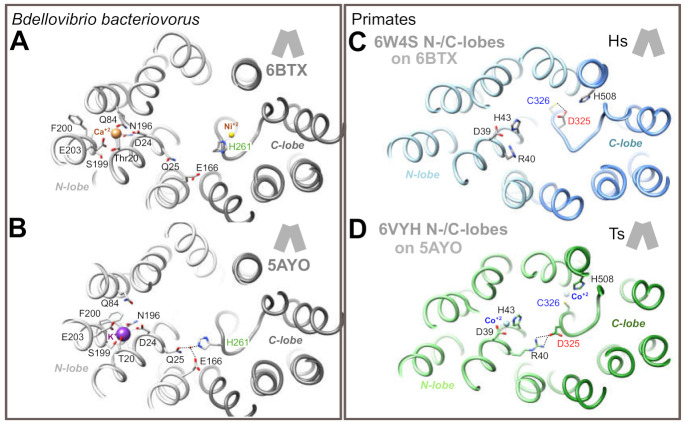
Predicted arrangement of the N- and C-lobes of the HsFPN1/TsFpn in an inward-facing conformation (panels **C** and **D**), as deduced from superimposition of the N- and C-lobes of the HsFPN1/TsFpn in an outward-facing conformation onto the corresponding lobes in the 3D structures of BbFpn solved in an inward-facing conformation (panels **A** and **B**). H-bonds and salt-bridges are shown with dashed lines. The BbFpn/TsFpn 3D structures also illustrate the position and features of the ion binding sites in the N-lobes.

We next evaluated the situation of BbFpn His261 and TsFpn/HsFPN1 Asp325 in the IF state. In this state, the N- and C-lobes come in close proximity to one another on the extracellular side, with a particular shift of TM7b toward the center of the architecture. However, this conformation has only been solved for BbFpn (Figure 4A,B), for which two 3D structures differ in that His261 is involved either in metal binding in the C-lobe (Figure 4A), or found in another conformation with its side-chain in a cationic imidazole state, in an opposite direction not able to bind metal (Figure 4B), similarly to what is observed in the OF state (Figure 3E,F). In the second situation (Figure 4B), the His261 side-chain is linked through a water molecule to the N-lobe Gln25 and Glu166 side-chains [19]. To model the possible position of HsFPN1 and TsFpn Asp325 in the IF state, we fitted each lobe of the HsFPN1 and TsFpn 3D structures in OF state (Figure 3A,C) onto the corresponding lobes of the BbFpn 3D structures in IF state, as depicted in Figure 4A (pdb 6BTX [19]) and Figure 4B (pdb 5AYO [18]). The rationale for this rigid-body reconstruction was based on the rocker-switch model followed by BbFpn between the OF and IF states and on the strikingly similar conformation of TM7, including the unwound segment (compare Figure 3D to Figure 3E,F). The N- and C-lobes of HsFPN1/TsFpn taken from the OF states globally superimposed well with those of BpFpn in the IF state, with a slight deviation on the extracellular side of the TM1-TM2 pair, consistent with gating helix rearrangements from each of the bundles. Comparison of the two primate architectures in the IF conformation showed that the Asp325 side-chain at 180° in TsFpn (when Co^2+^ is bound to Cys326; Figure 4D) relative to its position in the HsFPN1 model (when Asp325 is hydrogen-bonded to Cys326; Figure 4C) may form a salt-bridge with side-chain atoms of Arg40 in the N-lobe.

Taken together, these observations suggest that BbFpn His261 and HsFPN1/TsFpn Asp325 are critical actors for interaction with the N-lobe in the IF state, while the role of the two residues relative to the metal binding site in the C-lobe may differ between species, with Asp325 being a critical amino acid in the environment shaping metal binding by Cys326.

## 3. Discussion

As stated in the Introduction, the “rocker-switch” model is not sufficient to account for the different states occurring during conformational transitions in MFS transporters. Rather than symmetrical rocking of two structurally similar N- and C-terminal lobes and consequently only three expected conformations (outward-facing, occluded, and inward-facing), many MFS transporters have at least five distinct conformations: outward-facing, outward-occluded, occluded, inward-occluded, and inward facing [9,10]. The partially occluded states are induced by local changes in TMs referred to as “gating helices”, which block the substrate from exiting, but the MFS transporter is yet to undergo the global rocker-switch transition to its opposite-facing conformation [25]. In many cases, the substrate appears to bind evenly to both lobes, yet either the N-terminal lobe or the C-terminal lobe is thought to contribute more to the opening dynamics, thanks to flexible helices. In some cases, the substrate gating helices are easy to ascertain, as they are broken and contain unwound regions that connect to half-helices. A well-studied example is that of Glucose Transporters (GLUTs), which have a broken TM7 helix, with a short TM7b segment playing a key role in extracellular gating, even being directly involved in substrate preference through specific changes in gating dynamics [10,25]. A similar topology is observed for TM7 in the C-lobe of ferroportin [18,19,20,21], although here the exact coupling mechanism between iron binding and extracellular gating is unclear. The present study provides insights into the role that the negatively charged Asp325 residue may play in this context, at the bottom of FPN1 TM7b.

Previous studies demonstrated loss of iron efflux of the Asp325Ala, His, and Asn mutants, using different strategies and cell models (^55^Fe efflux in oocytes, ^55^Fe intracellular retention in HEK293T cells) [16,19]. Bonaccorsi di Patti et al. first indicated that the Asp325Ala mutant significantly reduced the iron transport ability of human FPN1. Western blot analysis and fluorescent microscopy data were provided in their Supplementary Materials to comment on the cellular distribution of this particular mutant, but the data were somewhat contradictory and not fully informative [16]. Deshpande et al. confirmed the profound impact of the Asp325Ala mutant on cellular iron export, making a comparison with the Asp325His mutant. This contrasted with the lowest effect, attributed to the change of aspartic acid 325 for asparagine. Live-cell imaging of oocytes over-expressing human FPN1 were presented in supplements. This was sufficient to ascertain that the Asp325 mutants were not absent from the plasma membrane of the transfected oocytes, but again there was some degree of uncertainty about the actual levels of the Asp325Ala mutant on the cell surface as compared to the wild-type protein [19]. The results we present here confirm the inability of the Asp325Ala, His, and Asn mutants to export iron in a manner similar to that observed for the wild-type protein, with a milder effect attributed to Asp325Asn (Figure 2). They also clearly demonstrate that the two Asp325Ala and Asp325His substitutions result in a dramatic decrease in cell-surface expression of the iron exporter, in contrast to Asp325Asn (Figure 1).

Models of HsFPN1 and TsFpn arrangement in an IF state suggest that, in addition to its role in the iron binding site within the C-lobe of human FPN1, the Asp325 side-chain shifts to form an inter-lobe salt-bridge with Arg40 from TM1 (Figure 4D). This salt-bridge holding the N- and C-lobes together may be linked to the presence of a metal ion in the C-lobe site, as it could not be formed in a metal-free OF state where the Asp325 rotamer is hydrogen-bonded to Cys326 (Figure 4C). In this case, Asp325 is likely to play a critical role in tuning an optimal local environment around Cys326, the SH group of which donates hydrogen bonds to the Asp325 carboxylate, while Asp325 is likely to allow Cys326 sulfur valence electrons to be polarized optimally for metal binding [26,27]. It thus seems likely that the negatively charged Asp325, which interacts only indirectly with Co^2+^ (in contrast to Cys326 and His507), plays an important role in repositioning TM7b on substrate binding. Substitution of Asp325 by an alanine or a histidine cannot maintain the predicted non-covalent interactions, either with TM7 (with Cys326) or with TM1 (with Arg40). The situation appears less deleterious with an asparagine, which maintains 1 oxygen atom and should still be able to interact with Cys326 and/or Arg40. The results presented in Figure 1; Figure 2 suggest that the Asp325-Arg40 inter-lobe interaction could be a key in the dynamics of the human FPN1 extracellular gate and in the stability of the iron exporter at the cell surface, whereas the Asp325-Cys326 interaction is also very important from a functional point of view but rather less essential, as is also suggested by the absence of an effect of mutants at position Cys326 on the iron export function [17,20,22]. Further investigations using MD simulations of the proposed IF architecture and/or experimental data are needed to clarify the details of the extracellular gate, in particular the local arrangement of the extracellular parts of TM1-TM2, on top of the Arg40-Asp325 bridge, in contact with TM7b.

How intracellular iron catalyzes local rearrangements within the human FPN1 C-lobe is an open question. It is useful here to recall that many MFSs utilize the so-called “proton-motive force” to drive the transport process [28,29], and that there is often a pair of glutamates, aspartates, or histidines, which are able to protonate or deprotonate in the translocation pathway, inside the central cavity of the MFS and in the vicinity of the substrate binding site [28]. This probably includes FPN1 in its IF conformation when the cytoplasmic iron comes to be exported, as very recently suggested by various groups using proteoliposome systems [21,29]. Whether FPN1 is a symporter (Fe^2+^ and H^+^ transported in the same direction) or an antiporter (Fe^2+^ and H^+^ transported in opposite directions) has not be fully elucidated, but it is important to also remember that cells have a negative resting membrane potential, making export of positively charged Fe^2+^ and H^+^ problematic energetically; thus, coupled import of protons is more likely to facilitate iron export. Pan et al. thus proposed a transport scheme in TsFpn where H^+^ and Fe^2+^ share binding sites but do not occupy them simultaneously. In their model, each transport cycle exports one Fe^2+^ that binds sequentially to the N-lobe (in a site containing the Asp39 and His43 residues) and the C-lobe (in a site containing the Cys326 and His508 residues) and imports two H^+^ that occupy both sites [21]. However, it is not necessary for protonation to be fixed at two identical positions during the OF-to-IF transition, provided that there is one of the titratable residues inside the central cavity assuming the protonation status at any given time. A proton may potentially be transferred between two binding sites according to solvent accessibility and the pKa values of the titratable sites [28]. In the Escherichia coli lactose permease (LacY), the most extensively studied prokaryotic MFS symporter, translocation of a galactoside across the membrane has been found to rely on proton binding to critical carboxylates, whereas dissociation is based on competitive binding of the cationic side chain of a neighboring basic residue. The position of these proton-interacting carboxylates is therefore very precise, and crucial for transport activity [30]. A counterexample is the multidrug transporter from Lactococcus lactis, which acts as a calcium/proton antiporter. This active proton-coupled MFS transporter exhibits remarkable plasticity in proton interactions, due to intrinsic flexibility in the location of key acidic residues within the central cavity that are part of two distinct substrate binding sites and are responsible for proton transfer [31]. In the Escherichia coli proton-coupled sugar symporter XylE, various studies suggested that proton binding at residue Asp27 in the OF conformation triggers extracellular closing. The proton then travels to one of the conserved acidic residues of the cytoplasmic networks, contributing to disruption of the inner gate and promoting the opening of the protein on the intracellular side. Direct interactions between the charge networks and surrounding lipids facilitate substrate release into the cytosol [32]. These are just some examples showing that substrate binding, proton coupling, and gating interactions in different conformational states differ subtly between MFSs, with local conformational changes overlapping with global ones to fine-tune substrate recognition and translocation. Further studies are needed to decipher the molecular details of proton-coupled iron transport in human FPN1, and the particular influence of protonation on rearrangements of the extracellular substrate gating the TM7b helix during the IF-to-OF transition.

To conclude, this study opens up novel perspectives on the molecular mechanisms governing iron export in humans, highlighting the complex and key role that Asp325 in TM7b may play in an inward-facing situation at the level of the extracellular gate, through a possible salt-bridge with TM1 Arg40. Hence, Asp325 may be a critical actor of the extracellular gate, before transition to an intermediate occluded state. This role is highly sensitive to occupancy of the C-lobe site by a metal ion. In addition, as shown by our various Asp325 mutants, the region of TM7 surrounding this amino acid is also important for stability of human FPN1 within the lipid bilayer. Taken together, this extends previous findings of intra- and inter-lobe salt-bridges on the cytoplasmic side the N-lobe (TM2, TM3, TM4, TM5) and C-lobe (TM10, TM11), which play a critical role in the organization and dynamics of the intracellular gate [20,33,34].

## 4. Materials and Methods

### 4.1. Plasmid Constructs

The wild-type SLC40A1-V5 plasmid construct was generated by cloning full-length human SLC40A1cDNA (GenBank accession number NM_014585.5) into the pcDNA3.1-V5-His-TOPO vector (Invitrogen, Life Technologies, Carlsbad, CA, USA). The same strategy was adopted to generate the HLA(A)-V5 plasmid construct. All ferroportin variations were introduced in the pcDNA3.1-SLC40A1/V5 vector using the QuikChange Site-Directed mutagenesis kit according to the manufacturer’s instructions (Agilent Technologies, Santa Clara, CA, USA). Sequencing analyses were performed to check the integrity of all the plasmid constructs.

### 4.2. Culture and Transfection of Human Epithelial Kidney (HEK)293T Cells

HEK293T cells from the American Type Culture Collection were incubated at 37 °C in 5% CO2 humidified atmosphere and propagated in Dulbecco’s Modified Eagle Medium (DMEM; Lonza, Newington, NH, USA) supplemented with 10% fetal bovine serum (Eurobio). Cells were transiently transfected using jetPEI (Polyplus transfection) according to the manufacturer’s recommendations, with a 2:1 transfection reagent (µL)/plasmid DNA ratio (µg).

### 4.3. Isolation of Cell-Surface Proteins and Western Blot Analysis

Membrane proteins were biotinylated and purified using the Pierce Cell Surface Protein Isolation Kit according to the manufacturer’s instructions (Pierce, Thermo Scientific, Rockford, IL, USA). Western blot analysis was performed using a mouse monoclonal primary antibody against V5 (Invitrogen) and then a polyclonal goat anti-mouse immunoglobulin/HRP (Dako) as secondary antibody. The membranes were incubated with the Immobilon Forte Western HRP substrate according to the manufacturer’s instructions (Merck Millipore, Burlington, MA, USA) and digitized for pattern analysis using the GeneGnome system (Syngene), Paris, France).

### 4.4. Densitometry Study

To quantitate ferroportin cell surface expression, the optic densities of SLC40A1/V5 and HLA-A/V5 protein bands were measured from the digital images by the GeneTools software (Syngene). Two normalizations were performed. First, ferroportin expression was normalized with cotransfected HLA(A). Second, WT ferroportin expression was set at 100%, and the expression of ferroportin variants was normalized to WT ferroportin expression.

### 4.5. ^55^Fe Release Measurements

55Fe loading of human apotransferrin was performed as previously described [35]. For iron release experiments, a modification of the protocol described by Schimanski et al. was used [36]. Briefly, HEK293T cells were seeded at 1.7 × 105 cells per well in 12-well plates, grown for 24 h in supplemented DMEM, and transfected with wild-type or mutated fpn-GFP plasmid constructs for 24 h before loading with 20 μg/mL 55Fe-transferrin for 16 h. Cells were washed once with PBS and cultured in Pro293a-CDM serum-free medium (Biowhittaker, Walkersville, MD, USA) for up to 36 h. 55Fe exported into the supernatant was collected at various time points, mixed with liquid scintillation fluid (ULTIMA GOLD MV_Packard Bioscience, Waltham, MA, USA) and counted for 2 min in a TRI-CARB 1600 CA scintillation counter (Packard, Waltham, MA, USA). Percentage 55Fe export was calculated using the following formula: (55Fe in the supernatant at 36 h, divided by cellular 55Fe at time zero) × 100.

### 4.6. 3D Structure Analysis

3D structures were visualized and analyzed using the UCSF Chimera program [37]. Coordinates of the experimental 3D structures were extracted from PDB (https:www.rcsb.org) and correspond to (i) BbFpn in an outward-facing state, in presence of Fe^+2^ (N-lobe, 5AYM) or of K^+^ (N-lobe, pdb 5AYN), (ii) BbFpn in an inward-facing state in presence of K^+^ (N-lobe, pdb 5AYO), (iii) BbFpn in an inward-facing state in presence of Ca^+2^ (N-lobe) and Ni^+2^ (C-lobe) (pdb 6BTX), (iv) Hs FPN1 in an outward-facing state, in an apo state (pdb 6W4S), or in presence of Co^+2^ and hepdicin (C-lobe, pdb 6WBV) and (v) Ts FPN in an outward-facing state, in presence of Co^+2^ (N- and C-lobes, pdb 6VYH) or in the presence of hepcidin (pdb 8WIK). The models of the possible architecture of Hs FPN1 in an inward-facing state were made considering the N- (aa 17-237/aa 17-238) and C-lobes (aa 17-238/aa 291-546) extracted from the 3D structures of Hs FPN1 in an outward-facing state (pdb 6VYH and 6W4S, respectively). These lobes were superimposed on the N and C-lobes of the BbFpn in an inward-facing states (pdb 5AYO and 6BTX, respectively).

## Data Availability

Not applicable.

## Abbreviations

MFS	Major Facilitator Superfamily
OF	Outward-facing
IF	Inward-facing
HsFPN1	Human Ferroportin
BbFpn	Bdellovibrio bacteriovorus Ferroportin
TsFpn	primate Philippine Tarsier Ferroportin
